# A Novel “Reactomics” Approach for Cancer Diagnostics

**DOI:** 10.3390/s120505572

**Published:** 2012-05-02

**Authors:** Sofiya Kolusheva, Rami Yossef, Aleksandra Kugel, Nirit Hanin-Avraham, Meital Cohen, Eitan Rubin, Angel Porgador

**Affiliations:** 1 Ilse Katz Institute for Nanoscale Science and Technology, Ben Gurion University of the Negev, Beer Sheva 84105, Israel; E-Mails: kolushev@bgu.ac.il (S.K.); nirithanin@yahoo.com (N.H.-A.); 2 The Shraga Segal Department of Microbiology and Immunology and the National Institute for Biotechnology in the Negev, Ben Gurion University of the Negev, Beer Sheva 84105, Israel; E-Mails: yossefra@bgu.ac.il (R.Y.); sasha.kugel@gmail.com (A.K.); kupervaser@yahoo.com (M.C.); erubin@bgu.ac.il (E.R.)

**Keywords:** biomarker, cancer, serum, polydiacetylene, diagnostics, bioinformatics

## Abstract

Non-invasive detection and monitoring of lethal diseases, such as cancer, are considered as effective factors in treatment and survival. We describe a new disease diagnostic approach, denoted “*reactomics*”, based upon reactions between blood sera and an array of vesicles comprising different lipids and polydiacetylene (PDA), a chromatic polymer. We show that reactions between sera and such a lipid/PDA vesicle array produce chromatic patterns which depend both upon the sera composition as well as the specific lipid constituents within the vesicles. The chromatic patterns were processed through machine-learning algorithms, and the bioinformatics analysis could distinguish both between cancer-bearing and healthy patients, respectively, as well between two types of cancers. Size-separation and enzymatic digestion experiments indicate that lipoproteins are the primary components in sera which react with the chromatic biomimetic vesicles. This colorimetric reactomics concept is highly generic, robust, and does not require *a priori* knowledge upon specific disease markers in sera. Therefore, it could be employed as complementary or alternative approach for disease diagnostics.

## 1. Introduction

Mortality rates of many cancers have not changed dramatically since the initiation of the “war on cancer” more than 30 years ago. Cancer detection and monitoring are considered as effective factors for improving cancer treatment and survival [1]. Hence, identification of novel tumor biomarkers and development of diagnostics technologies are critical constituents in the fight against cancer [1]. Cancer biomarker research generally focuses on blood as a non-tumoral surrogate tissue for cancer diagnostics. The continuous contact between the blood and the evolving cancer tissue gives rise to changes in blood molecular patterns originating either directly from the tumor or induced by the cancerous state. Accordingly, varied technology-based “omics” approaches—proteomics, metabolomics, glycomics, and others—have been proposed, so far with limited success, for identifying cancer patterns in blood components, such as cells, serum, or plasma [2–4]. Indeed, it has become clear that varied biological, physiological, and technical parameters significantly complicate biomarker discovery and validation, and often lead to “false discovery” [2–4].

This study describes a radically different approach for cancer (and other disease) diagnostics. Specifically, instead of trying to identify novel cancer biomarkers in sera, we focus here on the reactions of sera with an array of artificial biomimetic membrane detectors, a concept denoted reactomics. Essentially, our approach aims to exploit variations in sera content between cancer-bearing and healthy control patients for cancer diagnosis, through monitoring the interactions of the sera with arrays of vesicles containing lipid molecules and polydiacetylene (PDA), a chromatic polymer [5,6].

PDA is a conjugated polymer which exhibits unique color and fluorescence properties. In particular, we have shown over the past several years that the polymer matrix in lipid/PDA vesicle assemblies undergoes dramatic color transformations, accompanied by fluorescence changes that are induced by external stimuli—particularly interactions with soluble amphiphilic or membrane-active molecules [7]. In essence, in such PDA-based platforms, the conjugated polymer acts as a built-in reporter of lipophilicity and membrane affinity of soluble molecules, measurable by a chromatic change in both the visible absorption and fluorescence emission spectra. In the context of sera-membrane interactions, the chromatic signals induced by lipophilic components within sera constitute the fundamental means for distinguishing between normal and cancer conditions. Recently we have shown that lipid/PDA vesicles undergo chromatic transformations induced by lipoproteins extracted from blood sera [8]. In particular, the extent of chromatic transitions was shown to vary between lipoproteins separated from sera of healthy individuals and diabetic patients [8].

## 2. Experimental Section

### 2.1. Serum Harvesting, Handling, and Processing

Sera were obtained from RNTech Company (Paris, France). Fifty sera samples from pre-operation stomach cancer patients, 50 samples from pre-operation pancreatic cancer patients and 50 sera samples from cancer-free controls were studied. Clinical details are described at Supplemental Table 1. RNTech has established and conducted its activity following regulatory and ethical standards, implementing local, national, European, US and International (UN) rules and recommendations particularly when applicable to biological material collection and treatment and research result exploitation. These include both written consent of each patient contributing to the biological and data bank, and written study authorization from ethical committees of each clinical institute contributing samples to the company's biobank.

Sera from cancer patients and cancer-free controls were taken after overnight fasting in the following manner: 5 mL of blood was drawn into a vacuette serum tube (Cat# 456005, Greiner Bio One, Kremsmuenster, Austria) and left to clot for about 30 min, after which the tube was centrifuged at 3,000 rpm on a Hettich EBA 20S centrifuge (Hettich Ag, Tuttlingen, Germany) for 5 min at room temperature. The separated serum was aliquoted into 1 mL aliquots in sterile cryogenic tubes (Nalgene, Rochester, NY, USA) and immediately frozen at −70 °C. Sera samples were then transported on dry ice and stored at −70 °C immediately upon arrival. Sera samples were thawed on ice for about an hour and a half, 50 μL was aliquoted into lo-bind tubes (Eppendorf, Hamburg, Germany) and immediately re-frozen at −70 °C. All sample aliquots were stored at −70 °C until further processing (F2 freezing). For collecting 100 kDa serum retentate, two F2 aliquots (100 μL) were thawed on ice. 100 kDa centricons (YM-100, Millipore_TM_, Cat# 42413) were washed twice with 200 μL of TRIS buffer 50 mM-pH 7.2, and 90 μL thawed serum were loaded and centrifuged for 90 min at 4 °C at 5,000× g. Retentate was washed once on the centricon with 400 μL of TRIS buffer, diluted to twice the original serum sample volume (180 μL) with TRIS buffer, and freezed (F3 freezing) for future application to experimental plates with chromatic vesicles.

### 2.2. Lipids and Detector Chromatic Vesicle Preparation

1,2-Dimyristoyl-*sn*-glycero-3-phosphocholine (DMPC), 1,2-dioleoyl-*sn*-glycero-3-phosphocholine (DOPC), 1,2-dimyristoyl-*sn*-glycero-3-phosphoethanolamine (DMPE), 1,2-dioleoyl-*sn*-glycero-3-phosphoethanolamine (DOPE), 1,2-dimyristoyl-*sn*-glycero-3-phospho-(1′-rac-glycerol) (DMPG), L-α-phosphatidylserine (brain, porcine) (PS), L-α-phosphatidylinositol (liver, bovine) (PI), cardiolipin (heart, bovine) (CL), sphingomyelin (brain, porcine) (SM) and cholesterol (bovine wool) (Chl) were purchased from Avanti (Alabaster, AL, USA). The diacetylenic monomer 10,12-tricosadiynoic acid (PDA) was purchased from Alfa Aesar (Karlsruhe, Germany). The diacetylene powder was washed in chloroform and purified through a nylon 0.45 μm filter (Whatman) before use. Tris(hydroxymethyl)aminomethane (TRIZMA base buffer, C_4_H_11_NO_3_) was purchased from Sigma.

Chromatic vesicles containing the diacetylene monomer 10,12-tricosadiynoic acid and the lipid components (Table 1) were dissolved in chloroform/ethanol (1:1) and dried together *in vacuo* to constant weight, followed by addition of deionized water to a final concentration of 1 mM and subsequently probe sonicated at 40 W at 70 °C for 3 min. The vesicle solution was subsequently cooled at room temperature and kept at 4 °C overnight. The solution was then irradiated at 254 nm for 30 s, resulting in intense blue color appearance due to polymerization of the diacetylene units.

### 2.3. Chromatic Measurements: Fluorescence Spectroscopy

Fluorescence was measured on a Fluscan Ascent using a 96-well microplate (Greiner plate Cat# 655–180), using excitation of 544 nm and emission of 620 nm using LP filters with normal slits. Using this excitation/emission pair assured that the background fluorescence of the detector vesicle solutions before addition of the tested serum was negligible. Samples for fluorescence measurements were prepared by adding 5 μL processed serum to 30 μL of lipid/PDA detector vesicles followed by addition of 30 μL 50 mM Tris buffer (pH is depicted at Table 1). The samples were incubated for 60 min at 27 °C prior to measurements. Sixty min time point was chosen as the optimal time in which the chromatic response equilibrates (Figure S1). Fluorescent chromatic responses were calculated according to the formula: percentage fluorescent chromatic responses (%FCR) = [(Em_i_ − Em_c_)/(Em_r_ − Em_c_)] × 100%, in which Em_c_ is the background fluorescence of blue vesicles without addition of tested sample, Em_i_ is the value obtained for the vesicle solution after incubation with tested sample and Em_r_ is the maximal fluorescence value obtained for the red-phase vesicles (heating at 80 °C for 2 min). The result taken for each serum sample-specific detector was the mean of the triplicate.

### 2.4. Statistical Analysis

Experiments were performed in 96-well plates; a typical plate employed one type of detector vesicle and contained replicates of serum samples from each studied group as well as positive and negative color controls and identical aliquots of five standardization serum samples. Average %FCR per each sample was calculated based on the plate negative and positive color controls (see above, chromatic measurements: fluorescence spectroscopy). The %FCR values from different experimental plates were standardized according to the results of the five standardization serum samples employed in all experimental plates. To further correct for experimental biases between different experimental plates, a normalization step was applied to %FCR values in each experimental plate as follows: the mean %FCR of the experimental plate control serum samples was subtracted from each %FCR value and the result was divided by the standard deviation of the experimental plate control serum samples. This process was repeated for each chromatic vesicle, and each normalized %FCR was used as a feature in subsequent classification experiments. Classification was conducted using the support vector machine (SVM) method with a linear kernel as implemented in the LIBSVM library [9,10]. Separate machine learning experiments were conducted for each pair of class groups: Control *vs.* Stomach; Control *vs.* Pancreas and Pancreas *vs.* Stomach. The samples were randomly divided into training and testing subsets, maintaining the ratio of control cases to treatment cases analyzed in each experiment. For feature selection, all possible subsets were considered. An SVM model was developed for every possible subset of features, and the best model was chosen based on its accuracy of predicting the class of the training subset samples. The accuracy of this model was evaluated over the remaining testing group, using the percent of accurate prediction (“Accuracy”) and Mathews Correlation Coefficient (MCC) as quality measures. This procedure was repeated five times, using different random partitions into training and test sets each time, and the quality measures (classification Accuracy and MCC) were calculated for all partitions. For a binary classification test, Sensitivity measures the proportion of actual positives which are correctly identified as such and Specificity measures the proportion of negatives which are correctly identified. Accuracy is the proportion of true results (both true positives and true negatives) in the population. MCC is used in machine learning as a measure of the quality of binary (two class) classifications and returns a value between −1 and +1. A coefficient of +1 represents a perfect prediction, 0 an average random prediction and −1 an inverse prediction. MCC is generally regarded as a balanced measure which can be used even if the classes are of different sizes.

## 3. Results and Discussion

### 3.1. Fundamentals of the Reactomics Method

**Figure 1.**
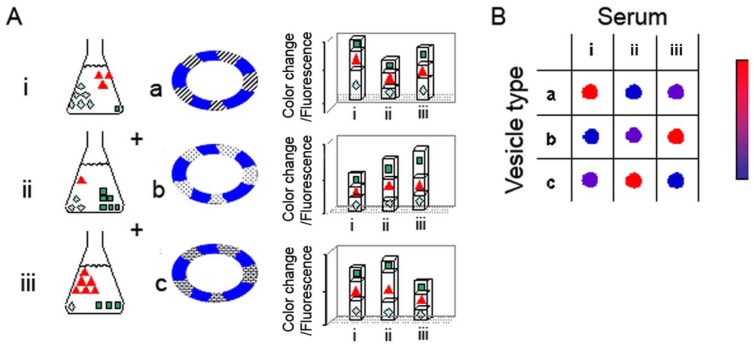
Schematic description of the *reactomics* concept. (A) Three tested sera, having varying compositions (i–iii), are applied to three vesicle types comprising PDA (blue), and different lipid compositions (a–c). The chromatic responses induced by the three sera in each vesicle are shown in the bar diagram; (B) The chromatic matrix depicting the relative degrees of chromatic response (color/fluorescence) in the sera/vesicle assembly tested in (A). Each serum is assigned a distinct “chromatic pattern” depending upon its content of vesicle-reactive species on the one hand and the lipid composition of the vesicles on the other hand.

The hypothesis underlying the reactomics approach is that molecular variations of sera associated with cancer onset and progression provide a window of opportunity for disease detection and monitoring. The diagnostic concept and experimental concept are depicted schematically in Figure 1. Figure 1(A) represents a generic experiment in which three sera are examined (sera i–iii), using an array of three lipid/PDA vesicle compositions (vesicles a–c); the actual experiments we carried out (see below) employed a larger array of lipid/PDA vesicles. Each serum examined (represented by i–iii) can be perceived as a mixture of varied amphiphilic/vesicle-active species. Accordingly, upon interactions with a particular lipid/PDA vesicle, the serum produces a chromatic signal which is essentially a sum of the contributions of all individual components in the mixture.

As depicted in the schematic picture in Figure 1, vesicle variability is the core feature facilitating the diversity of signals generated in the chromatic system. Essentially, the sera are applied to an array of lipid/PDA vesicles comprising PDA and different lipid molecules (chromatic vesicles a–c). Each serum is expected to induce a distinct chromatic (color/fluorescence) transition when added to a particular lipid/PDA vesicle. Importantly, the total color/fluorescence transformations will depend upon the distinct affinities of sera components to lipids having different structures, head-group charges, membrane packing, and other molecular properties. Overall, application of each serum sample to the vesicle array will result in a chromatic pattern (each row in Figure 1(B)), in which the number of components is determined by the different vesicle compositions employed in the experiment. Crucially, through application of simple bio-informatics algorithms, we show here that distinct color patterns (e.g., chromatic fingerprints) can be discerned following interactions between sera from cancer-bearing and healthy individuals, respectively, and the lipid/PDA vesicle array. We show here that these disease-marker patterns were statistically distinguishable from the patterns recorded for healthy patients.

Previous studies have shown that PDA-based vesicle assays can be carried out in specific pH “windows”; in solutions exhibiting pH under 6.5 the PDA matrix does not undergo chromatic transitions, while at highly basic solutions (generally pH > 9–9.5), PDA changes its color/fluorescence due to the high concentration of the hydroxide ions. In the experiments depicted here we have optimized the pH conditions individually for each vesicle composition, accounting for the different environmental sensitivity of each composition. The pH values ranged between 7.5–8.5, and with most samples around 8 (Table 1).

### 3.2. Vesicle Activity of Sera and the Molecular Components Affecting Chromatic Transitions

**Figure 2.**
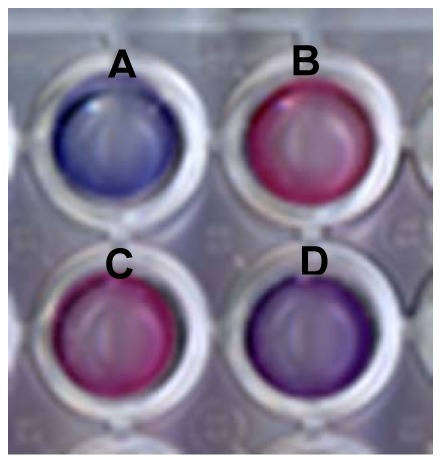
Color transitions in lipid/PDA vesicles induced by serum. DMPC/PDA vesicle solutions are shown prior/after incubation for 30 min with human sera. (A) Control solution (no addition of serum); (B–D) vesicles were incubated with sera obtained from different samples.

Figure 2 depicts the colorimetric transformations observed upon incubation of lipid/PDA vesicles with sera. The scanned picture in Figure 2 clearly shows that DMPC/PDA vesicles that were initially blue underwent noticeable color changes upon incubation with different sera. Importantly, Figure 2 indicates that changes in sera-induced chromatic transitions were apparent between serum obtained from healthy individuals (Figure 2(B,C)) and a cancer-bearing patient (Figure 2(D), serum sample from stomach cancer patient). However, some variations in chromatic transitions were also observed between the color transitions induced by sera from healthy persons (Figure 2(B) *vs*. (C)). These variations were the impetus for the comprehensive statistical method, described below, which was designed to distinguish and correlate among sera and pathological conditions.

**Figure 3.**
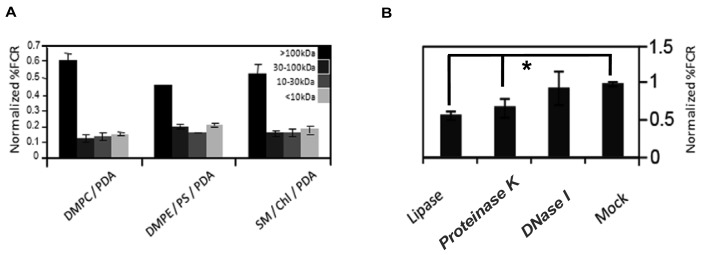
Effects of size fractionation and enzyme treatment upon the chromatic reactions of serum with lipid/PDA vesicles. (A) Using centricons, serum was continuously fractionated to >100 kDa, 30–100 kDa, 10–30 kDa and <10 kDa fractions. Aliquots from each fraction, derived from the same serum quantity, were reacted with three different chromatic vesicles and the sum of %FCR reactions of all fractions per each chromatic vesicle was normalized to one; (B) Identical aliquots of serum were treated with the different enzymes or with mock treatment and reacted with DMPC/PDA (2:3 mole ratio). Proteinase K and Lipase by themselves did not affect the basic chromatic response. Similar results were observed with five other lipid/PDA vesicles. Experiments were performed in triplicates and the results were normalized to the reaction with mock treated-serum. ** *p-value* < 0.01, single factor ANOVA.

To partially characterize the vesicle-reactive species in serum, we size-separated serum components employing Centricon filtration, and separately carried out an enzymatic digestion assay (Figure 3). Serum samples were separated to >100 kDa, 30–100 kDa, 10–30 kDa, and <10 kDa, respectively, and the size-separated fractions were subsequently incubated with chromatic vesicles having three different lipid compositions (Figure 3(A)). Figure 3(A) clearly shows that the primary response for all chromatic vesicles was manifested by the >100 kDa fraction, which included high-molecular weight proteins but also serum nanoparticles like lipoproteins. Indeed, we previously showed that the chromatic vesicles exhibited significant chromatic response when incubated with purified lipoproteins [8]. Furthermore, differences in vesicle binding between low-density lipoproteins (LDL) and high-density lipoproteins (HDL) purified from sera were correlated with physiological conditions such as diabetes [8].

To further test the assumption that lipoproteins are primary contributors to the reaction of serum with the chromatic vesicles we recorded the fluorescence changes undergone by the vesicles following digestion with different enzymes ((Figure 3(B)). Specifically, we treated the serum with DNase, protease, or lipase, which degrade a broad substrate scope of DNA, proteins, and lipids, respectively. Figure 3(B) shows that treatment of serum with DNase did not affect serum interactions with the chromatic vesicles, while digestion of the serum with lipase or protease considerably reduced the chromatic response (Figure 3(B)). While the data in Figure 3(B) cannot rule out that individual lipid and protein molecules in serum contributed to the chromatic vesicle signals, the results in both Figure 3(A,B) suggest that lipoproteins are plausible candidates for the primary vesicle-active components in serum. Indeed, the lipophilicity of lipoprotein surface could constitute the driving force for vesicle surface binding and the chromatic interactions. This hypothesis was further corroborated through the observation that serum-derived lipoproteins concentrated through sodium borate-based centrifugation, induced significant chromatic response when added to lipid/PDA vesicles (data not shown). Lipoproteins are composed of a lipid core and surface-displayed proteins, in which apolipoproteins are the primary component. The notion that apolipoproteins' levels (and thus lipoproteins) in blood are potential biomarkers for different cancers was recently reported [11,12]. Indeed, ApoC-I was identified as a potential serum biomarker for colorectal cancer, hormone-refractory prostate cancer, and liver fibrosis [12–14]. Other reports indicated that ApoC-III might also be a potential biomarker in pancreatic cancer and breast cancer [11,15].

Finally, we tested the sensitivity and repeatability of the assay for serum samples. When purified lipoproteins were applied in a dose response assay to the chromatic vesicles (e.g., DMPC/PDA), a %FCR range between 20 to 80 was reached upon incubation with 50 to 250 μg protein/mL of purified HDL [8]. When control sera were applied to the chromatic vesicles, a similar response range was reached upon incubation with 1 to 10 μL sera (data not shown). Therefore, we employed the equivalent of 5 μL serum volume per each chromatic vesicle in the following experiments. To test repeatability, five control serum samples were tested in a 10-repeat assay with the different chromatic vesicles (Figure S2). For eight of the ten chromatic vesicles, average relative standard deviation (RSD) for the five control sera was ≤8. For DOPC/PDA and DMPE/PS/PDA vesicles (Table 1), average RSD was 12.6 and 11.4, respectively. Overall, these RSD values represent good repeatability.

### 3.3. Chromatic Experiments of Sera from Cancer-Bearing and Healthy Control Groups

Serum sample aliquots from 50 subjects were analyzed per each studied clinical group: stomach cancer, pancreatic cancer, and non-cancer controls. Information on the sera samples and their physiological profiles is provided in the Supplementary Information. The vesicle array we employed contained ten different types of chromatic detector vesicles (Table 1), designed to span a broad range of lipid properties, including head group size and charge, alkyl-chain saturation, and transition temperatures [16].

**Figure 4.**
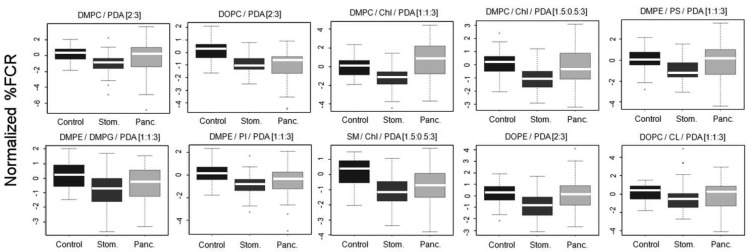
Chromatic reactions induced by the incubation of sera samples with ten different chromatic vesicles. Sample size is 50 per each clinical group and specific chromatic vesicle. The header of each panel indicates the composition of the chromatic vesicle used. Results are presented as standard box and whiskers plots of the normalized %FCR (see Methods). Briefly, for each distribution the main box depicts the 1st (bottom) and 3rd (top) quartiles, the band inside the box depicts the median, and the whiskers depict the upper and lower extreme values that are within 1.5 times the inter-quartile range. Control correspond to chromatic response of the vesicles following addition of sera from *healthy* individuals, stom. corresponds to sera from stomach-cancer patients, panc. corresponds to sera from pancreatic-cancer patients.

The vesicles were incubated with 100 kDa-separated serum sample aliquots from the clinical groups and percentage fluorescent chromatic responses (%FCR) were calculated per each plate based on the negative and positive color controls. Subsequently, %FCR results from different experimental plates were standardized and normalized to adjust for experiment-specific biases (see Methods). Figure 4 shows the normalized %FCR results per each chromatic vesicle type on the 3 group classes; results are presented as standard box and whiskers plots (see legend).

Differences between patterns of chromatic responses were clearly observed in Figure 4. Specifically, sera from stomach cancer patients induced chromatic change to a lesser extent as compared to sera from the control group. This result was very encouraging as we already reported for the same sera samples that both mass-spectrometry based peptidomics and clinical-based apolipoprotein analysis pointed to reduced quantities of apolipoproteins C-I and C-III in sera derived from stomach cancer patients [17]. Combining this observation with the results from Figure 3 the reported interaction of the chromatic vesicles with lipoproteins [8], it is evident that the correctness of the reactomics approach results is supported by results derived from other technologies. Sera from pancreas cancer patients showed a mixed chromatic response as compared to control. While the median of chromatic reaction was higher as compared to control with DMPC/Chl/PDA (1:1:3) and DMPE/PS/PDA [1:1:3] chromatic vesicles, sera from pancreas cancer induced chromatic change similarly or to a lesser extent as compared to control with the other eight types of vesicles. However, the lack of clear straightforward differences and the large margins of distribution (Figure 4) support the need for machine learning-based protocols for the production of prediction classifiers.

### 3.4. Bioinformatics Analysis of Chromatic Data Reveals Patterns that Distinguish between the Different Clinical Groups

To test the ability of the chromatic array to identify cancer patients and to distinguish pancreatic cancer from stomach cancer, we used machine learning algorithms to develop disease classifiers of the various studied groups. In this approach, chromatic responses are considered to be *features*, and a learning algorithm uses a training set (*i.e*., examples) to identify a complex set of rules, or a model, that is further used to classify new samples (test set) given the same features. To do that we randomly divided the 50 samples from each class group to form balanced training and test sets (*i.e*., 25 samples per each set, per each class group). We chose to use the support vector machine (SVM) algorithm for its robustness and strength. However, because SVM is a binary classification algorithm, separate machine-learning experiments were performed for each pair of class groups: (i) control *vs.* stomach cancer; (ii) control *vs.* pancreatic cancer; and (iii) pancreatic cancer *vs.* stomach cancer. The processed chromatic result from each specific detector vesicle (Figure 4) was employed as a feature for the SVM. Therefore chromatic results from 10 different detector vesicles (Table 1) yielded 10 features per tested sample.

Building a successful classifier using machine learning usually requires that an informative subset of features is selected for model development (*i.e.*, feature selection). Due to the relatively small number of features involved in this study (10 features), all the possible combinations of features could be considered (n = 1,023). In other words, every possible combination of features involving 1–10 features was tested for the training set. Essentially, for each features subset, an SVM model was trained and evaluated using only the training subset. The classifier that gave the highest Accuracy and Matthews Correlation Coefficient (MCC) values was chosen (see Methods for definitions). These measures of classifier quality reflect the concordance between predicted and actual classes of the samples (e.g., control or stomach cancer); however, while the Accuracy value reports only the fraction of correct predictions, the MCC statistic also accounts for the frequency of each class in the original sample, and is thus a more robust measure of prediction quality. The quality of the model that gave the highest Accuracy and MCC in the training set was then evaluated by applying it to new samples, namely the test set, and evaluating its Accuracy and MCC measures in this test set. Table 2 shows these parameters for the test set.

To further ensure that the predictive efficiency of the chromatic response–based classifier was not due to a fortuitous partition to training and testing subsets, we repeated the partition process 5 times, choosing for each repeat the best features subset (*i.e*., the best model) based on the training set and then applied the best model to the test set. The Accuracy and MCC values for the test set (Table 2) did not noticeably vary between repeats, ranging from 84.3% to 90.2% (or an MCC of 0.70–0.81) for the pancreatic cancer *vs.* control classification. This range of accuracy values is well within the 95% confidence interval from mean accuracy of the five repeats (estimated with the binomial distribution). Similarly, the range of accuracies observed from the stomach cancer *vs.* controls and stomach cancer *vs.* pancreatic cancer, respectively, also fall within the confidence interval of the mean accuracy observed in the five repeats.

The accuracies achieved by the reactomics-based classifier analysis, depicted in Table 2 for the test set, were significantly better than expected through random selection (e.g., “chance” selection). For the comparison of pancreatic cancer *vs.* controls, an accuracy of 84% or better was obtained for the testing group for all five repeats (Table 2); the likelihood of achieving such classification accuracy even *once* by chance is very low (p = 7 × 10^−7^, binomial distribution), let alone five times. The comparison of stomach cancer and pancreatic cancer patients yielded accuracy levels ranging from 71% to 81%; similarly, the likelihood to obtain 71% accuracy by chance even once is less than 3 × 10^−4^.

Table 2 indicates that distinguishing between stomach cancer sera and controls was less convincing than the two other pairs, with estimated accuracy levels ranging from 62.8 to 72.6%. Yet the likelihood to obtain 62.8% is less than 0.02, let alone repeating such a result or better five times. Overall, the reactomics-based detection of pancreatic cancer state was better compared to stomach cancer. In particular, the prediction's specificity was lower when comparing stomach cancer to either control or pancreas cancer (Table 2). This could be attributed to the fact that some detector vesicles manifested enhanced reaction with the pancreatic cancer sera as compared to control and other detectors manifested the opposite trend. Yet, sera from stomach cancer patients generally manifested a reduced reaction as compared to control sera (Figure 4). Our results can only hint to the ability of this approach to distinguish cancer types: the distinction between healthy controls and pancreatic cancer was better than the classification of healthy controls *vs.* stomach cancer. Without investigating additional tumor types, it is difficult to predict which types will be amenable to detection with the reactomics approach, and which can be distinguished from one another. The results presented here were based on 10 lipid compositions. It is tempting to speculate that finer classification can be obtained by adding additional compositions to obtain more sensitive and informative patterns. With such improved sensitivity, it might be possible to achieve higher classification accuracies, better patient stratification and to assist in the diagnosis of a wider spectrum of tumors. Indeed, we are currently investigating additional compositions of detector vesicles.

It is important to note that the statistical analyses described above did not involve intensive multiple testing: while over a thousand models were considered for the training set, the chosen model was then tested only once with the test set. The successful classification of pre-operation sera from pancreatic cancer and stomach cancer patients has two additional implications. First, it excludes the possibility that being at pre-operative state is the main factor mediating the differences revealed by the reactomics approach. Secondly, it suggests that sera derived from patients with different cancer diseases could manifest a specific reactomics pattern.

To further validate the statistical significance of the results, a shuffling experiment was conducted in which the class names of the different samples were randomly rearranged. Specifically, the 150 samples were randomly assigned 50 “Control” labels, 50 “stomach cancer” labels and 50 “pancreatic cancer” labels. The entire binary classification process, from partitioning to model building, through feature selection, up to evaluation on a test set was then repeated with the shuffled data. Crucially, the MCC of the best classifiers generated with the shuffled data was close to 0, which would be expected from a classifier that performs as well as guessing. This result clearly demonstrates the validity of the reactomics analysis and indicates that the “true” clinical assignment of the sample was essential for the generation of an accurate classification model (data not shown).

Three classification experiments were conducted, comparing pancreas cancer *vs.* control patients, stomach cancer *vs.* control patients, or stomach cancer *vs.* pancreas cancer patients. Each classification experiment involved partitioning the data into training and testing subsets, selecting the most informative features for the training set and evaluating the quality of the resulting model predicting class of the individuals in the testing subset and comparing the results to their actual classes. The process was repeated five times (rep. #), randomly choosing training and testing subsets for each repeat. Since the entire process was repeated, the features selected in each repeat were not always the same (Selected Features). Features that were selected in all five repeats for a given classification experiments are underlined (consensus features). Table 1 shows the description of the corresponding detector vesicle. The accuracy of each model was evaluated with the appropriate testing subset, using both the Accuracy parameter, determined by Sensitivity and Specificity, and the Mathews Correlation Coefficient (MCC) measures (see Methods for definitions). It is interesting to note that the “consensus features” of each replicate was not reported as the best classifier of any of the replicates. Because every possible feature combination was considered, the implication is that without exception, the consensus set did not perform better than the reported feature set in the training set in any of the replicates (Table 2). It is possible that the consensus set would produce more stable, general models that would perform better in the testing set. However, it is important to note that using the stability of features across five replicate experiments “contaminates” the test set, as samples from the test set of one replicate would be included in the training set of another, and vice versa. Despite this bias toward better performance in the testing set of “consensus features”, the chosen models generally outperform the consensus features set in the five replicates (data not shown).These results may suggest that adding features beyond the “consensus set” produces models with better accuracy, but that the added feature' contribution is small and is thus unstable across replicates. This hypothesis can be tested by considering additional samples.

Our results indicate that reactomics can be used to distinguish cancer patients from healthy individuals and these different patterns can be identified in the sera of patients harboring different tumors. However, further research is required to evaluate the power of this approach for early diagnosis. The majority of the pancreatic cancer samples, which were successfully classified, came from patients with stage I–II tumors (45 of 50 samples). Nevertheless, for screening purposes very high level of accuracies must be attained. The number of samples analyzed with this approach will have to be much higher to allow the false-positive rate to be estimated with sufficient accuracy to imply clinical usefulness. For stomach cancer, on the other hand, only half of the samples were from early stages (25 of the 50 samples were from stages I–II), making it impossible to estimate the potential of our approach for early detection. Further stratifying these patients, not only by tumor stage but by survival, requires that larger patient populations be investigated.

## 4. Conclusions

In our study we show that chromatic patterns produced through interactions between blood sera and an array of lipid/PDA vesicles containing different lipid compositions, constitute an effective vehicle for distinguishing between cancer-bearing and healthy patients. Furthermore, statistical analysis showed that the chromatic data can be used to discern between different cancers. Overall, the novel “reactomics” concept is a promising tool for disease diagnostics through pattern analysis.

## Supplementary Material



## Figures and Tables

**Table 1.** Lipid and PDA compositions of the detector vesicles.

**No.**	**Composition**	**Mole ratio**	**pH**
**1**	DMPC/PDA	2:3	8
**2**	DOPC/PDA	2:3	7.4
**3**	DMPC/Chl/PDA	1:1:3	8
**4**	DMPC/Chl/PDA	1.5:0.5:3	8
**5**	DMPE/PS/PDA	1:1:3	8
**6**	DMPE/DMPG/PDA	1:1:3	8
**7**	DMPE/PI/PDA	1:1:3	8
**8**	SM/Chl/PDA	1.5:0.5:3	8.2
**9**	DOPE/PDA	2:3	7.6
**10**	DOPC/CL/PDA	1:1:3	7.8

Abbreviations are explained in the Methods. pH of each vesicle solution was set in order to equilibrate the intrinsic sensitivity.

**Table 2.** SVM-based classification of cancer patients from serum reactome measurements.

**Rep. #**	**Selected Features**	**Accuracy**	**Sensitivity**	**Specificity**	**MCC**
**Pancreatic cance *vs*. control**					
1	No. 1, 2, 3, 4, 5, 8, 10	90.20	84.62	96.00	0.81
2	No. 1, 2, 3, 4, 7, 8, 10	86.27	80.77	92.00	0.73
3	No. 1, 2, 3, 6, 7, 10	90.20	88.46	92.00	0.80
4	No. 1, 2, 3, 5, 8, 10	86.27	80.77	92.00	0.73
5	No. 1, 2, 3, 9, 10	84.31	76.92	92.00	0.70

**Stomach cancer *vs.* control**					
1	No. 2, 4, 6, 7, 9	68.63	84.62	52.00	0.39
2	No. 1, 2, 4, 5, 7, 8, 9, 10	62.75	65.38	60.00	0.25
3	No. 2, 6, 9	70.59	80.77	60.00	0.42
4	No. 1, 2, 4, 7, 8, 9	70.59	65.38	76.00	0.42
5	No. 2, 3, 4, 6, 7, 8	72.55	69.23	76.00	0.45

**Stomach cancer *vs.* pancreatic cancer**					
1	No. 1, 2, 3, 4, 7, 9	80.77	84.62	76.92	0.62
2	No. 1, 2, 3, 6, 7, 9	78.85	84.62	73.08	0.58
3	No. 1, 2, 3, 4, 5, 9	76.92	84.62	69.23	0.54
4	No. 1, 2, 3, 6, 7, 9	71.15	76.92	65.38	0.43
5	No. 1, 3, 4, 5, 8, 9, 10	76.92	73.08	80.77	0.54
