# Supplementary material for: A Novel “Reactomics” Approach for Cancer Diagnostics
Source: Sensors (Basel). 2012 May 2;12(5):5572–85. doi: 10.3390/s120505572 (PMC3386700; doi:10.3390/s120505572)
Supplement: Supplementary file 1 [file sensors-12-05572-s001.pdf]

OPEN ACCESS

*sensors*

ISSN 1424-8220

www.mdpi.com/journal/sensors

*Supplementary Information*

# A Novel "Reactomics" Approach for Cancer Diagnostics. *Sensors* 2012, *12*, 5572-5585

**Sofiya Kolusheva [1,†], Rami Yossef [2,†], Aleksandra Kugel [2], Nirit Hanin-Avraham [1], Meital Cohen [2], Eitan Rubin [2] and Angel Porgador [2,*]**

[1] Ilse Katz Institute for Nanoscale Science and Technology, Ben Gurion University of the Negev, Beer Sheva 84105, Israel; E-Mails: kolushev@bgu.ac.il (S.K.); nirithanin@yahoo.com (N.H.-A.)

[2] The Shraga Segal Department of Microbiology and Immunology and the National Institute for Biotechnology in the Negev, Ben Gurion University of the Negev, Beer Sheva 84105, Israel; E-Mails: yossefra@bgu.ac.il (R.Y.); sasha.kugel@gmail.com (A.K.); kupervaser@yahoo.com (M.C.); erubin@bgu.ac.il (E.R.)

† These authors contributed equally to this work.

* Author to whom correspondence should be addressed; E-Mail: angel@bgu.ac.il; Tel.: +972-8-647-7283; Fax: +972-8-647-7626.

---

**Table S1.** Age distribution, gender and clinical characteristics of sera samples used. All samples were collected and stored at Fundeni Clinical Institute Bucharest, Romania by RNTech Company. Samples were shipped to Ben-Gurion University, Israel and processed following established protocols (see Methods). Abbreviations: AJCC/UICC TNM classification, American Joint Committee on Cancer (AJCC)/International Union against Cancer (UICC) tumor-node-metastasis (TNM) classification.

| Group | Age at Excision | Sex | Clinical Diagnosis (specimen) | AJCC/UICC TNM classification | AJCC/UICC Stage Group |
|---|---|---|---|---|---|
| Stomach | 75 | F | Adenocarcinoma | T2bN2M0 | IIIA |
| Stomach | 63 | M | Adenocarcinoma | T2bN0M0 | IB |
| Stomach | 55 | M | Adenocarcinoma | T2aN0M0 | IB |
| Stomach | 64 | F | Adenocarcinoma | T2bN2M1 | IV |
| Stomach | 58 | M | Adenocarcinoma | T4N2M0 | IV |
| Stomach | 70 | F | Adenocarcinoma | T3N2M0 | IIIB |
| Stomach | 67 | M | Adenocarcinoma | T2aN0M0 | IB |
| Stomach | 66 | F | Adenocarcinoma | T2aN1M0 | II |
| Stomach | 66 | F | Adenocarcinoma | T2bN1M0 | II |
| Stomach | 59 | M | Adenocarcinoma | T2aN2M0 | IIIA |
| Stomach | 63 | F | Adenocarcinoma | T2AN1M0 | II |

**Table S1.** *Cont.*

| Group | Age at Excision | Sex | Clinical Diagnosis (specimen) | AJCC/UICC TNM classification | AJCC/UICC Stage Group |
|---|---|---|---|---|---|
| Stomach | 61 | M | Adenocarcinoma | T2bN1M0 | II |
| Stomach | 65 | F | Adenocarcinoma | T2aN1M1 | IV |
| Stomach | 62 | M | Adenocarcinoma | T2aN2M0 | IIIA |
| Stomach | 65 | M | Adenocarcinoma | T3N3M0 | IV |
| Stomach | 65 | M | Adenocarcinoma | T2bN0M0 | IB |
| Stomach | 74 | M | Adenocarcinoma | T2bN2M0 | IIIA |
| Stomach | 75 | F | Adenocarcinoma | T2bN1M0 | II |
| Stomach | 68 | M | Adenocarcinoma | T3N1M0 | IIIA |
| Stomach | 63 | M | Adenocarcinoma | T3N2M0 | IIIB |
| Stomach | 69 | M | Adenocarcinoma | T2bN1M0 | II |
| Stomach | 72 | M | Adenocarcinoma | T2bN2M0 | IIIA |
| Stomach | 66 | M | Adenocarcinoma | T2bN2M0 | IIIA |
| Stomach | 63 | M | Adenocarcinoma | T2bN1M0 | II |
| Stomach | 63 | M | Adenocarcinoma | T2bN1M0 | II |
| Stomach | 69 | M | Adenocarcinoma | T2bN1M0 | II |
| Stomach | 59 | M | Adenocarcinoma | T4N2M0 | IV |
| Stomach | 60 | M | Adenocarcinoma | T2aN2M0 | IIIA |
| Stomach | 70 | F | Adenocarcinoma | T2aN1M0 | II |
| Stomach | 66 | M | Adenocarcinoma | T2bN1M1 | IV |
| Stomach | 59 | M | Adenocarcinoma | T2aN1M0 | II |
| Stomach | 57 | F | Adenocarcinoma | T2bN0M0 | IIA |
| Stomach | 64 | M | Adenocarcinoma | T2bN1M0 | II |
| Stomach | 45 | M | Adenocarcinoma | T4N2M0 | IV |
| Stomach | 69 | M | Adenocarcinoma | T2aN2M0 | IIIA |
| Stomach | 63 | M | Adenocarcinoma | T2bN2M0 | IIIA |
| Stomach | 65 | M | Adenocarcinoma | T2bN0M0 | IIA |
| Stomach | 53 | M | Adenocarcinoma | T2bN0M0 | IIA |
| Stomach | 67 | M | Adenocarcinoma | T2N1M0 | II |
| Stomach | 68 | M | Adenocarcinoma | T2aN0M0 | IIA |
| Stomach | 57 | M | Adenocarcinoma | T3N2M0 | IIIB |
| Stomach | 59 | F | Adenocarcinoma | T3N1M0 | IIIA |
| Stomach | 56 | F | Adenocarcinoma | T2bN3M0 | IV |
| Stomach | 56 | M | Adenocarcinoma | T2aN0M0 | IIA |
| Stomach | 59 | M | Adenocarcinoma | T3N2M1 | IV |
| Stomach | 77 | M | Adenocarcinoma | T2bN1M0 | II |
| Stomach | 65 | M | Adenocarcinoma | T2aN0M0 | IIA |
| Stomach | 71 | M | Adenocarcinoma | T3N2M1 | IV |
| Stomach | 66 | M | Adenocarcinoma | T3N2M0 | IIIB |
| Stomach | 52 | M | Adenocarcinoma | T3N0M0 | II |
| Pancreas | 64 | F | Adenocarcinoma | T1N1M0 | IIB |
| Pancreas | 69 | F | Carcinome | T3N0M0 | IIA |
| Pancreas | 58 | M | Carcinome | T1N1M0 | IIB |

**Table S1.** *Cont.*

| Group | Age at Excision | Sex | Clinical Diagnosis (specimen) | AJCC/UICC TNM classification | AJCC/UICC Stage Group |
|---|---|---|---|---|---|
| Pancreas | 72 | F | Adenocarcinoma | T3N0M1 | IV |
| Pancreas | 60 | M | Adenocarcinoma | T3N0M0 | IIA |
| Pancreas | 56 | F | Adenocarcinoma | T2N0M0 | IB |
| Pancreas | 59 | F | Adenocarcinoma | T1N1M1 | IV |
| Pancreas | 77 | M | Carcinome | T2N0M0 | IB |
| Pancreas | 69 | F | Carcinome | T3N1M0 | IIB |
| Pancreas | 55 | M | Carcinome | T1N1M0 | IIB |
| Pancreas | 52 | F | Carcinome | T2N0M0 | IB |
| Pancreas | 75 | M | Carcinome | T2N0M0 | IB |
| Pancreas | 64 | M | Carcinome | T2N0M0 | IB |
| Pancreas | 61 | F | Carcinome | T2N1M0 | IIB |
| Pancreas | 46 | M | Carcinome | T2N1M0 | IIB |
| Pancreas | 59 | F | Carcinome | T3N0M0 | IIA |
| Pancreas | 74 | M | ADK, benign disease | T2N0M0 | IB |
| Pancreas | 52 | F | Carcinome | T2N1M0 | IIB |
| Pancreas | 67 | F | Adenocarcinoma | T2N1M0 | IIB |
| Pancreas | 73 | F | Adenocarcinoma | T3N1M0 | IIB |
| Pancreas | 52 | M | Carcinome | T3N0M0 | IIA |
| Pancreas | 54 | F | Carcinome | T2N1M1 | IV |
| Pancreas | 62 | F | Carcinome | T2N1M0 | IIB |
| Pancreas | 65 | M | Adenocarcinoma | T3N0M0 | IIA |
| Pancreas | 67 | M | Adenocarcinoma | T2N1M0 | IIB |
| Pancreas | 57 | M | Adenocarcinoma | T2N1M1 | IV |
| Pancreas | 45 | M | Adenocarcinoma | T2N0M0 | IB |
| Pancreas | 67 | F | Carcinome | T4N0M0 | III |
| Pancreas | 53 | M | Adenocarcinoma | T2N1M0 | IIB |
| Pancreas | 64 | F | Adenocarcinoma | T1N0M0 | IA |
| Pancreas | 42 | F | Adenocarcinoma | T2N0M0 | IB |
| Pancreas | 54 | M | Adenocarcinoma | T3N1M0 | IIB |
| Pancreas | 59 | F | Carcinome | T1N0M0 | IA |
| Pancreas | 71 | M | Adenocarcinoma | T3N1M0 | IIB |
| Pancreas | 54 | M | Adenocarcinoma | T3N1M0 | IIB |
| Pancreas | 66 | F | Adenocarcinoma | T3N1M0 | IIB |
| Pancreas | 60 | M | Adenocarcinoma | T3N1M0 | IIB |
| Pancreas | 60 | M | Adenocarcinoma | T3N1M0 | IIB |
| Pancreas | 61 | M | Adenocarcinoma | T3N1M0 | IIB |
| Pancreas | 60 | M | Adenocarcinoma | T2N0M0 | IB |
| Pancreas | 61 | M | Adenocarcinoma | T3N0M0 | IIA |
| Pancreas | 53 | F | Adenocarcinoma | T2N0M0 | IB |
| Pancreas | 71 | M | Adenocarcinoma | T3N0M0 | IIA |
| Pancreas | 45 | M | Adenocarcinoma | T3N0M0 | IIA |
| Pancreas | 77 | F | Adenocarcinoma | T3N0M0 | IIA |

**Table S1.** *Cont.*

| Group | Age at Excision | Sex | Clinical Diagnosis (specimen) | AJCC/UICC TNM classification | AJCC/UICC Stage Group |
|---|---|---|---|---|---|
| Pancreas | 66 | F | Carcinome | T2N0M0 | IB |
| Pancreas | 72 | M | Adenocarcinoma | T3N0M0 | IIA |
| Pancreas | 51 | M | Adenocarcinoma | T2N0M0 | IB |
| Pancreas | 59 | M | Adenocarcinoma | T3N1M0 | IIB |
| Pancreas | 59 | F | Adenocarcinoma | T1N1M1 | IIB |
| Control | 65 | F | | | |
| Control | 68 | M | | | |
| Control | 75 | M | | | |
| Control | 65 | M | | | |
| Control | 66 | F | | | |
| Control | 66 | M | | | |
| Control | 66 | M | | | |
| Control | 70 | M | | | |
| Control | 69 | F | | | |
| Control | 67 | F | | | |
| Control | 76 | M | | | |
| Control | 67 | F | | | |
| Control | 71 | F | | | |
| Control | 70 | F | | | |
| Control | 58 | M | | | |
| Control | 69 | M | | | |
| Control | 73 | F | | | |
| Control | 65 | M | | | |
| Control | 72 | F | | | |
| Control | 56 | M | | | |
| Control | 34 | F | | | |
| Control | 28 | F | | | |
| Control | 27 | F | | | |
| Control | 28 | M | | | |
| Control | 27 | F | | | |
| Control | 26 | F | | | |
| Control | 27 | M | | | |
| Control | 31 | M | | | |
| Control | 29 | F | | | |
| Control | 28 | F | | | |
| Control | 19 | M | | | |
| Control | 30 | F | | | |
| Control | 40 | M | | | |
| Control | 32 | F | | | |
| Control | 43 | F | | | |
| Control | 27 | F | | | |
| Control | 26 | F | | | |
| Control | 40 | M | | | |
| Control | 30 | M | | | |
| Control | 28 | F | | | |
| Control | 29 | F | | | |

**Table S1.** *Cont.*

| Group | Age at Excision | Sex | Clinical Diagnosis (specimen) | AJCC/UICC TNM classification | AJCC/UICC Stage Group |
|---|---|---|---|---|---|
| Control | 30 | M | | | |
| Control | 36 | F | | | |
| Control | 30 | F | | | |
| Control | 26 | M | | | |
| Control | 33 | M | | | |
| Control | 29 | F | | | |
| Control | 40 | F | | | |
| Control | 33 | F | | | |
| Control | 26 | F | | | |

**Figure S1.** Representative measurement of %FCR following incubation of healthy donor-serum with chromatic lipid-PDA vesicles in different time points.

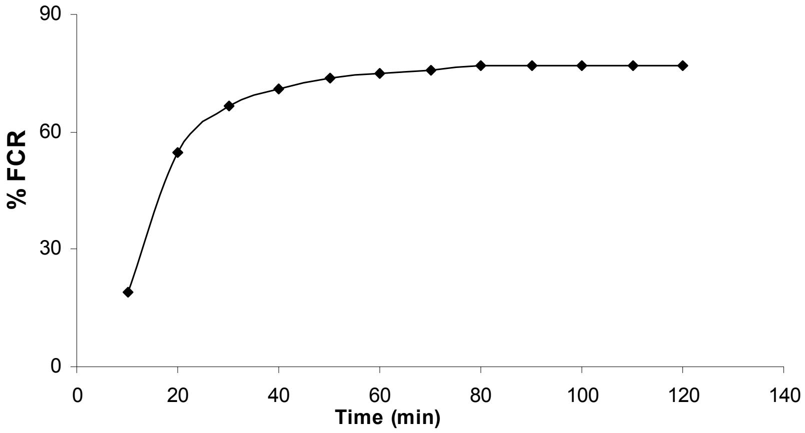


**Figure S2.** Five control sera (S1 to S5) were tested in a 10-repeat assay for the chromatic response induced by 10 different chromatic lipid-PDA vesicles (X-axis, see Table 1). For each serum and chromatic vesicle, RSD of the 10-repeats is presented and the black line represents the average RSD for the 5 sera.

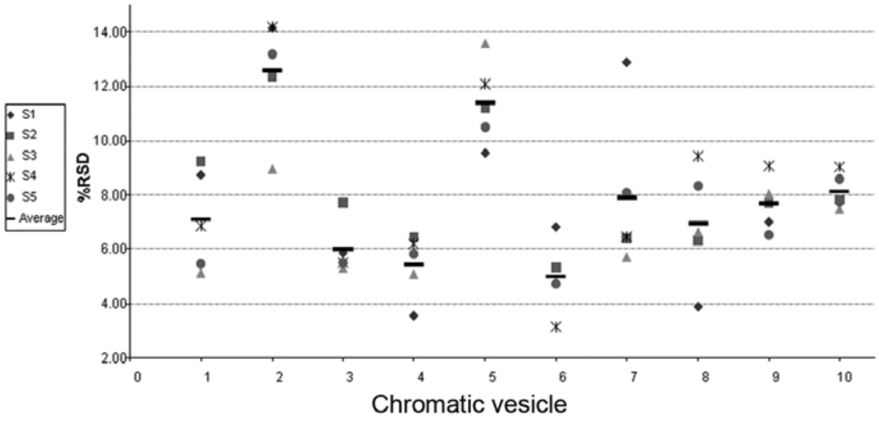


© 2012 by the authors; licensee MDPI, Basel, Switzerland. This article is an open access article distributed under the terms and conditions of the Creative Commons Attribution license (http://creativecommons.org/licenses/by/3.0/).
